# Longitudinal Changes in Daily Subjective Sleep and Age From the Everyday Function Intervention Trial

**DOI:** 10.1093/geroni/igaf122.825

**Published:** 2025-12-31

**Authors:** Hye Won Chai, Alyssa Gamaldo, Lesley Ross

**Affiliations:** Clemson University, Clemson, South Carolina, United States; Clemson University, Clemson, South Carolina, United States; Clemson University, Clemson, South Carolina, United States

## Abstract

Some cognitive training interventions, such as Useful Field of View training (UFOVt) are effective in maintaining brain health, cognition, and everyday functioning. However, daily changes associated with training are less understood. This study examined daily changes in sleep and subjective age as outcomes of UFOVt. Data came from the Everyday Function Intervention Trial (EFIT; NCT04651582) study, a randomized clinical controlled trial designed to assess the moderators and mechanisms of UFOVt transfer effects among older adults aged between 65 to 90 years (N = 94). EFIT participants completed laptop-based cognitive, health, and psychosocial surveys during baseline, post-test, and three-month follow-up as well as smartphone-based daily assessments throughout the study period. Data utilized for this study included daily assessments of subjective sleep (e.g., sleep quality, restfulness, difficulty falling asleep, difficulty staying asleep) and subjective age (e.g., “Compared to your actual age, what age do you feel right now?”) collected across 14 days at baseline, post-test, and follow-up, respectively (i.e., three 14-day bursts of daily data). Multilevel analyses assessed the longitudinal changes between baseline and follow-up in person-level average and fluctuations in daily sleep and subjective age, and whether these changes differ by training. Results demonstrated that UFOVt group reported reductions in difficulty falling asleep and in feeling older than their actual age compared to the active control group. These results suggest UFOVt as a potentially useful tool for improving older adults’ subjective assessment of their everyday experiences and well-being which may be protective of cognitive decline.

